# An interview with Philip Biggin, Section Editor for Computational, *in silico* and modelling studies

**DOI:** 10.1186/2050-6511-13-2

**Published:** 2012-08-13

**Authors:** Philip C Biggin

**Affiliations:** 1 Structural Bioinformatics and Computational Biochemistry Unit, Department of Biochemistry, University of Oxford, South Parks Road, Oxford, OX1 3QU, UK

## 

Phil Biggin, PhD, is a group leader in the Department of Biochemistry, University of Oxford. He is interested in computational approaches to receptor dynamics and ligand-binding. His group uses a wide range of methodologies ranging from molecular dynamics simulations through to machine learning techniques to address fundamental problems in pharmacology. He is the Section Editor for Computational, in silico and modelling studies for BMC Pharmacology and Toxicology. In this interview we find out a little more about the key issues in this field of research.

## How did you first become interested in pharmacology and toxicology?

I became interested in chemistry and computers as a teenager which led to my decision to read Computer-aided Chemistry for my first degree. During that time I spent a fascinating industrial year at SmithKline Beecham (now GSK) developing software to aid the computational chemistry group. Although at that time I did not know I wanted to do a PhD, I did know that I wanted to continue investigating the way in which drugs work. I then undertook a PhD in the biophysics of ion channels, which helped fill in some of the biological gaps in my knowledge. The rest is history as they say.

## Why is it an exciting time to be involved with computational aspects of pharmacology and toxicology in particular?

Computational chemistry and molecular modelling have been embedded within the drug-design process for many years now and indeed most pharmaceutical companies have some kind of computational chemistry group within their Research and Development programmes. From early quantitative structure activity relationship (QSAR) studies and visualization through to cheminformatics and molecular dynamics, the scope and power of these techniques is ever increasing and they are starting to yield important results that are not only interestingly academically, but can result in substantial financial savings for drug companies.

## What challenges and developments can we expect to see in the next few years?

One of the major challenges for computational chemists remains the prediction of affinity for small molecules with protein (and nucleic acid) targets. There have been many approaches to this ranging from thermodynamically rigorous free energy calculations through to machine learning methods. Although there have been examples of good success, the prediction of absolute affinities remains a major challenge for the field. If the prediction of affinity was not challenging enough, the next problem will be the prediction of efficacy. Just because a compound binds with high affinity does not necessarily mean it has high efficacy. In fact the problem may be even worse than that, because it is becoming apparent that some systems exhibit 'agonist bias' whereby the efficacy of the same agonist is not maintained across different pathways as discussed by Terry Kenakin in his review article on this topic [1]. These aspects will be particularly challenging because efficacy and agonist bias will encompass the dynamic behaviour of the protein in response to the ligand-binding event.

## How can computational modelling contribute to meeting these challenges?

Both of the problems above will become more tractable with physical models as the computational power available increases. In fact, it is already possible to explore timescales that are not only experimentally relevant but also able to address problems that are difficult if not impossible to address by experiment alone [2]. We can certainly expect to see a closer link between computational and experimental studies in that regard.

## How can computational models help in the prediction of adverse drug reactions?

Many of the challenges we face are still at the one protein-one drug level, but in order to predict pharmacology and toxicology in the future effectively, more consideration will have to be given to absorption, distribution, metabolism, excretion and toxicity (ADMET). Many adverse drug reactions are caused by unintended activity at off-targets. There has been some recent success in those types of predictions [3], and although experiments are still needed to verify the results, the predictions are useful enough to draw attention to possible problems earlier in the drug-discovery process, thus saving money. One might also expect protein structure and dynamics to play an increasing role in the prediction of ADMET properties [4] as it is quite often the case that off-target proteins have large flexible binding pockets, something, which until recently, has largely been ignored.

## Is there a role for computational modelling in the regulatory assessment of novel chemical entities?

The best predictions will be those that account for all of the effects of a compound within the body and to that end aspects of systems biology will certainly have a key role to play. Indeed, physiologically based pharmacokinetic (PBPK) modelling is already used to guide the decision-making process within regulatory review [5]. One might expect these kinds of models to be integrated with pharmacogenetics studies to make further improvements in the coming years.

## What does the future hold for computational pharmacology and what do you think are its limitations?

The quality of future predictions will be underpinned by two main factors: 1) Knowledge or data and 2) computing power. As both of those are rising exponentially, one can look forward to more accurate computational pharmacology sooner rather than later.

## Are there any particular papers you would like to see submitted to your section?

In keeping with the journal ethos to publish work deemed by peer review to be a coherent and sound addition to scientific knowledge I would be particularly interested in seeing papers on drug interactions with other macromolecular systems, especially those that are supported by experimental data and shed light on the underlying mechanism. Of course, that is a slightly personal view and we really would welcome submissions from across the whole field of Computational, *in silico* and modelling studies.

## Competing interests

Philip Biggin is a Section Editor on *BMC Pharmacology and Toxicology.*

## Authors’ contributions

PB wrote and approved the final text.

## Pre-publication history

The pre-publication history for this paper can be accessed here:

http://www.biomedcentral.com/2050-6511/13/2/prepub
